# Cell Type Resolved Expression of Duplicate Genes Retained From Whole Genome Duplication in Atlantic salmon

**DOI:** 10.1093/gbe/evaf076

**Published:** 2025-04-30

**Authors:** Richard S Taylor, Rose Ruiz Daniels, Daniel J Macqueen

**Affiliations:** The Roslin Institute and Royal (Dick) School of Veterinary Studies, The University of Edinburgh, Midlothian, UK; The Roslin Institute and Royal (Dick) School of Veterinary Studies, The University of Edinburgh, Midlothian, UK; The Roslin Institute and Royal (Dick) School of Veterinary Studies, The University of Edinburgh, Midlothian, UK

**Keywords:** whole genome duplication, single cell transcriptomics, duplicate genes, ohnologs, expression evolution, transcriptional responses, salmonid fish

## Abstract

The functional and evolutionary outcomes of whole genome duplication (WGD) events are driven by global remodeling of gene expression. Most investigations of gene expression changes following WGD have applied bulk transcriptomics using tissue samples, thus failing to resolve affected cell types. Here, we leverage single-cell transcriptomics of liver tissue in Atlantic salmon (*Salmo salar* L.) to quantify cell-specific expression and transcriptional responses to a bacterial infection with *Aeromonas salmonicida* for thousands of duplicate gene pairs (ohnologs) retained from WGD ancestral to all salmonids. The major liver cell types showed hundreds of differentially expressed ohnolog pairs, with hepatocytes showing the greatest number and immune cells the least number of uniquely differentially expressed pairs. Many more differentially expressed ohnolog pairs were identified after accounting for cell type heterogeneity within a cell lineage, despite a reduction in statistical power. The degree of conservation in ohnolog expression responses to bacterial infection also varied significantly among cell types, both in terms of the number of differentially expressed pairs and the direction of responses. Overall, this study highlights the importance of resolving cell-specific gene expression to understand the functional and evolutionary outcomes of WGD events.

**Significance**

Whole genome duplication (WGD) leads to large-scale changes in the expression of duplicated genes, which may promote evolutionary innovations. Many studies have investigated how the expression of duplicated genes change during evolution using complex tissue samples, providing an average representation of cell types present. Most studies have failed to resolve changes in the expression of duplicate genes in individual cells. In this study, we investigated the expression of thousands of duplicated genes retained from WGD in salmonid fishes, using thousands of single cells sampled from liver. We show that the expression of duplicate genes varies greatly across distinct cell types. Consequently, our results indicate that distinct evolutionary pressures have acted on different cell types following WGD.

## Introduction

Whole genome duplication (WGD) has occurred repeatedly during eukaryotic evolution and is widely considered a driver of genetic and phenotypic diversification (Ohno 1970; Freeling and Thomas 2006; Van de Peer et al. 2017; Moriyama and Koshiba-Takeuchi 2018). WGD creates duplicated genes (ohnologs) across the genome, which may be retained and acquire novel functions via several pathways (Ohno 1970; Conant and Wolfe 2008; Innan and Kondrashov 2010). Large-scale changes in gene expression and regulation often follow WGD events and may have contributed to evolutionary novelties, for instance, those defining the vertebrate lineage (Marlétaz et al. 2018).

Understanding how gene expression evolves is central to revealing the functional outcomes of WGD, with most studies approaching this problem using bulk transcriptomic tools like RNA-Seq (e.g. Lien et al. 2016; Pasquier et al. 2016; Qiao et al. 2019). Bulk transcriptomics captures gene expression averaged across all cell types in target samples, which fails to resolve cell-specific expression and may overlook contributions from rare cell types. Cell-resolved ohnolog expression has been investigated in a handful of studies using single cell transcriptomics tools, which are being rapidly uptaken in non-model species (Ruiz Daniels et al. 2023). This includes work in plants with a history of allopolyploidization (i.e. where different species hybridized before WGD, leading to 2 subgenomes), including *Arabidopsis thaliana*, *Zea mays* (maize), and *Triticum aestivum* (bread wheat). These studies showed that evolutionary divergence in ohnolog expression was strongly influenced by cell type (Coate et al. 2020; Guillotin et al. 2023; Zhang et al. 2023). Cell-speciﬁc divergence has been found between genes on different subgenomes in the allopolyploid goldﬁsh (Kon et al. 2022); however, to what extent these differences arose prior to hybridization or as a consequence of the genome doubling is unknown. Another study emphasized the importance of cell-specific expression evolution in ohnologs retained from WGD in the teleost fish ancestor (Shafer et al. 2022).

The ancestor to salmonids experienced WGD around 100 million years ago (Macqueen and Johnston 2014; Gundappa et al. 2022), with 55% to 60% of all genes retained in ohnolog pairs from this event (Lien et al. 2016). The salmonid WGD occurred in addition to the stem teleost WGD and is thought to have resulted from autopolyploidization based on several lines of evidence (Allendorf and Thorgaard 1984; Lien et al. 2016; Taylor et al. 2021; Gundappa et al. 2022). A substantial fraction of salmonid ohnologs have evolved divergent expression in different tissues according to bulk transcriptomics (Lien et al. 2016; Robertson et al. 2017; Gillard et al. 2021; Gundappa et al. 2022). Single-cell transcriptomics has revealed individual cases where salmonid ohnologs for specific genes show distinct expression across cell types, for example, in liver (Taylor et al. 2022). However, past work has failed to systematically resolve the importance of different cell types to ohnolog expression changes in salmonids. Here, we combined single-cell transcriptomics and comparative genomics to investigate global changes in salmonid ohnolog expression resolved to specific cell types, considering both baseline expression and responses to immunological stimulation, which are known to induce ohnolog-specific responses in bulk transcriptomics studies (e.g. Clark et al. 2023). Our findings reveal extensive cell-specific variation in ohnolog expression and demonstrate the value of single cell transcriptomics in providing a deeper understanding of genome functional evolution following WGD.

## Results

### Datasets Used to Compare Ohnolog Expression Across Liver Cell Types

**Fig. 1.**
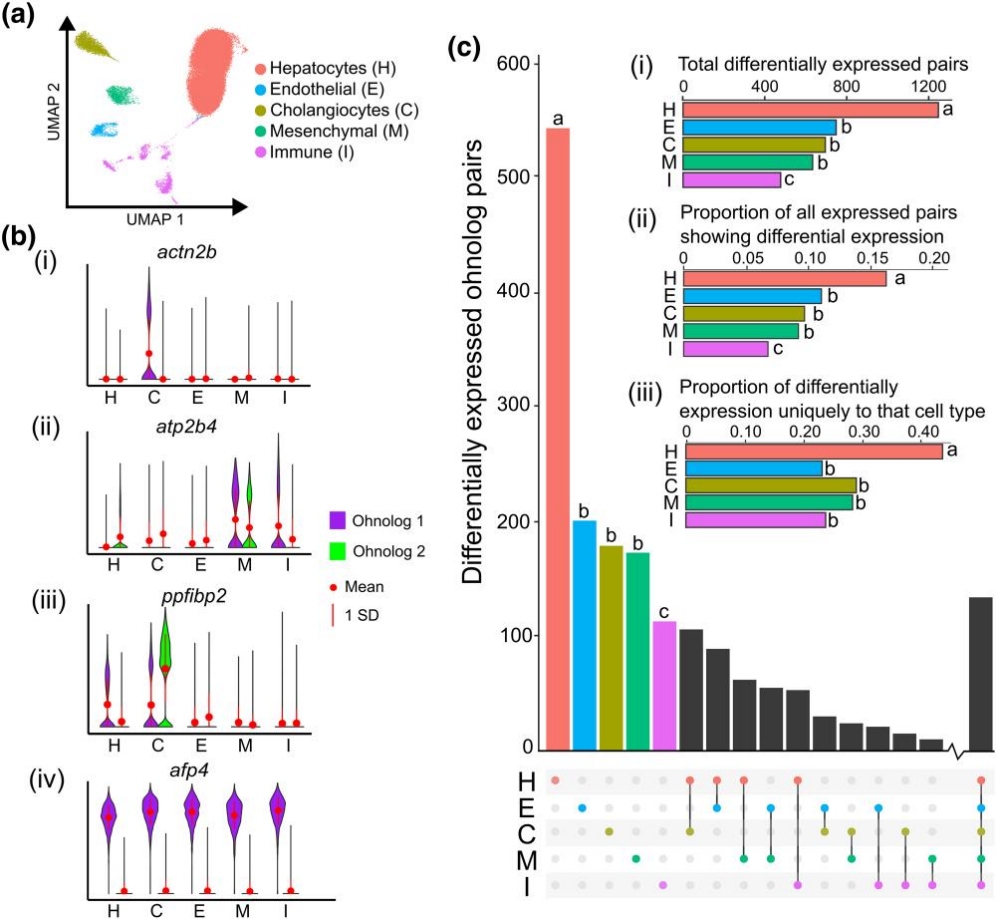
a) Single nuclei transcriptomes from healthy Atlantic salmon liver with assigned cellular identities (after Taylor et al. 2022). b) Four example ohnolog pairs are shown to illustrate the types of expression divergence observed across the major liver cell types. The scale shown for gene expression level represents log normalized UMI counts in each cell type. c) Upset plot showing both cell type–specific and cell type shared ohnolog pair expression level divergence across the 5 liver cell types. Three embedded bar chart panels show the total number of differentially expressed ohnolog pairs by cell type; the proportion of all expressed ohnolog pairs in each cell type that exhibit differential expression; and the proportion of all differentially expressed pairs that show differential expression only in the shown cell type. In all graphs, unique letters indicate statistically significant differences between cell types, while shared letters indicate non-significant differences, according to Fisher's exact test (see results in supplementary tables S1 to S4, Supplementary Material online).

We utilized an existing dataset comprising 47,432 nuclei transcriptomes from the liver of Atlantic salmon, including 2 control samples and 2 samples from fish challenged with the bacterial pathogen *Aeromonas salmonicida*. The nuclei transcriptomes were classified into 5 major cell lineages: hepatocytes, endothelial cells, mesenchymal cells, cholangiocytes, and immune cells (Taylor et al. 2022; Fig. 1a).

To investigate cell type specific variation in ohnolog expression, we cross-referenced all expressed genes in this dataset with a high-confidence set of salmonid ohnologs (Bertolotti et al. 2020). This homology prediction combined phylogenetic and syntenic information to identify high-confidence ohnologs retained from the salmonid WGD, as well as singleton genes, where one member of the ohnolog pair was lost during salmonid evolution.

Of the 47,329 coding genes annotated in the Atlantic salmon genome, 29,905 of these were expressed in at least one nuclei in this study. Of these, 17,090 genes were identified to be members of 8,545 ohnolog pairs arising from the salmonid-specific WGD (4R), defined to be pairs of genes arising from 4R with both copies still present in the Atlantic salmon genome. Five thousand five hundred and eighteen genes were identified to be singletons i.e. genes that were once a member of an ohnolog pair arising in 4R but one member of the pair was subsequently lost. This provides a robust foundation to explore the dynamics of ohnolog expression across different cell types.

### Differences in Ohnolog Expression Across Cell Types

Differential expression between ohnolog pairs was inferred across the major liver cell lineages using the control samples. To normalize statistical power, we downsampled the number of transcriptomes in each population to that of the cell type with the fewest nuclei. Disregarding cell type annotations, 1,594 (11.2%) ohnolog pairs showed significant differences in expression level (Wilcoxon rank-sum test, adjusted *P* < 0.05, log_2_ fold change > 0.25). To assess the power of our snRNA-seq data to identify differentially expressed ohnolog pairs in comparison to bulk transcriptomics, we performed a comparable analysis using bulk RNA-seq data from Atlantic salmon liver (*n* = 8; Gillard et al. 2021), which revealed a comparable 12.6% of ohnolog pairs to be differentially expressed. We then performed the same analysis for each of the 5 cell types from the snRNA-seq data, revealing 2,104 (14.8%) differentially expressed ohnolog pairs in at least one cell lineage (examples in Fig. 1b), an additional 510 pairs compared with the snRNA-seq analysis that ignored cell types.

Next, we compared the number of differentially expressed ohnolog pairs shared across all combinations of different cell types (Fig. 1c). Hepatocytes exhibited the highest absolute number of differentially expressed ohnologs and immune cells the fewest (Fig. 1c; supplementary tables S1 and S2, Supplementary Material online). To check that this result was not the consequence of different numbers of ohnolog pairs being expressed in each cell type, we confirmed that hepatocytes also had a significantly higher proportion (16.3%) of differentially expressed ohnolog pairs among all expressed ohnolog pairs compared with the other 4 cell types (Fig. 1c; supplementary table S3, Supplementary Material online). Immune cells also showed a significantly lower proportion (6.8%) of differentially expressed ohnolog pairs compared with the other 4 cell types (Fig. 1c; supplementary table S1, Supplementary Material online; supplementary table S3, Supplementary Material online). Additionally, hepatocytes demonstrated a significantly higher fraction (approximately 43%) of cell type-specific differential expression of ohnolog pairs than the other 4 cell types (Fig. 1c; supplementary table S4, Supplementary Material online).

For all cell types except immune cells, cell-specific differential ohnolog expression was the most prevalent category of differential expression (Fig. 1c; examples in Fig. 1b). A smaller yet substantial proportion of ohnolog pairs exhibited differential expression that was common to more than one cell type, with the most frequent category being those shared across all cell types. In these cases, the same ohnolog in each pair was consistently more highly expressed across all tested cell lineages (e.g. Fig. 1b, see *afp4*), indicating that the counterpart ohnolog was universally silenced in the liver. This contrasts with the situation where ohnolog pairs showed differential expression in only 2 to 4 cell types, when the same member of each pair could be either up- or down-regulated relative to its partner in each cell type.

### Cell-specific Expression of 4 *csf1r* Paralogs Arising From 2 Rounds of WGD

The cell-lineage labeled “Immune” (Fig. 1a) includes a diverse range of different cell types showing distinct expression profiles, with 13 different immune sub-populations identified in these data (Taylor et al. 2022) including 7 lymphocyte populations and 4 myeloid populations. The major classes of lymphocytes (B cells, T cells, natural killer cells) and myeloid cells (macrophages/monocytes, granulocytes, dendritic cells) have ancient origins in vertebrate evolution, with distinct gene expression programs that are well characterized in mammals and widely used to annotate cell types in single-cell studies. The expression of genes encoding colony-stimulating factor 1 receptor (*csf1r*) is commonly used as a marker for monocytes and macrophages within the myeloid lineage, which indeed informed a previous annotation of these cells in Atlantic salmon liver (Taylor et al. 2022). Given the evidence for extensive cell type specific differential expression identified earlier, we investigated the divergence in expression of all paralogous *csf1r* genes within myeloid cells annotated in the original publication, selected as an exemplar to infer impacts on the interpretation of single cell transcriptomic studies in salmonids.

**Fig. 2.**
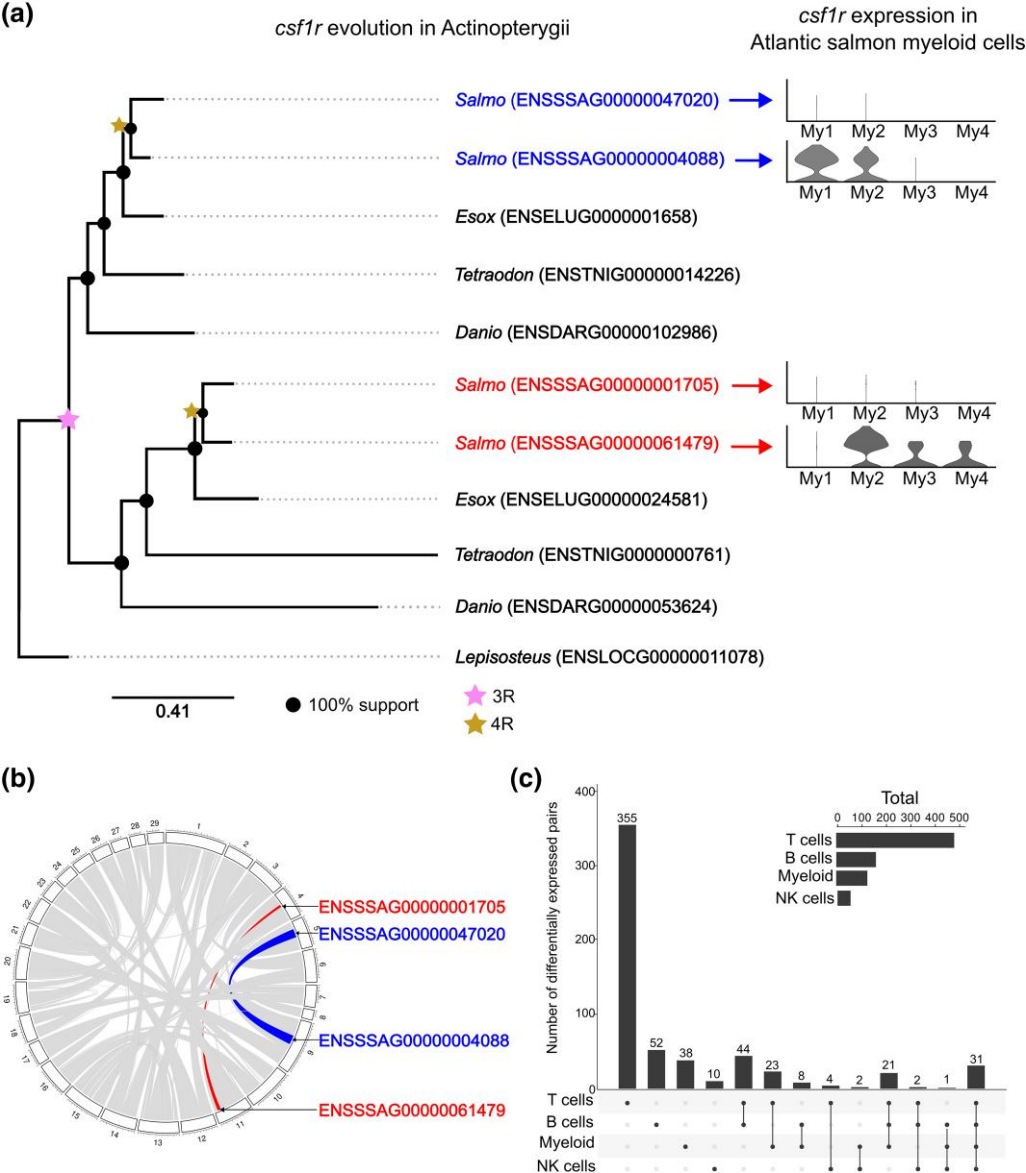
a) Phylogeny of colony stimulating factor 1 receptor (cfsr1) proteins in 5 Actinopterygii taxa: spotted gar (Lepisosteus oculatus; outgroup to 3R WGD; tree rooted to this species), zebrafish (Danio rerio), green spotted pufferfish (Tetraodon nigroviridis), northern pike (Esox Lucius; outgroup to 4R WGD) and Atlantic salmon *(Salmo salar*). Highlighted gene names denote ohnolog pairs arising from 4R. Expression levels of the 4 *csf1r* paralogs in Atlantic salmon are shown in 4 myeloid populations (My1-My4) previously identified in Atlantic salmon liver (Taylor et al. 2022). b) Genomic locations of the 4 *csf1r* genes in the Atlantic salmon genome. Bands represent collinear blocks retained from 4R WGD (Lien et al. 2016), with the location of csf1r genes colored by block. c) The number of ohnolog pairs exhibiting differential expression across the 4 major immune cell types in Atlantic salmon liver. Comparisons of numbers between the 4 immune populations should be avoided due to differences in statistical power arising from differing cell numbers in each population. The nuclei labelled “T cells” comprise 5 distinct subpopulations of T cells, while nuclei labelled “Myeloid” comprise 2 macrophage subpopulations, 2 dendritic cell sub-populations, and one neutrophil population (Taylor et al. 2022).

Four copies of *csf1r* were identified in the Atlantic salmon genome. Phylogenetic analysis was consistent with a single ancestral *csf1r* gene duplicating during the teleost-specific third round of WGD (3R) (Fig. 2a). 3R was elsewhere considered the likely origin of *csf1r* ohnologs in teleosts (Singh and Isambert 2019). The tree supports that the retained 3R ohnologs duplicated again during 4R leading to the 4 identified genes (Fig. 2a). This inference is further supported by the retention of *csf1r* pairs in large collinear blocks retained from 4R (Fig. 2b) (Lien et al. 2016; Gundappa et al. 2022).

Within the myeloid (My) compartment, expression of the 4 *csf1r* copies varied considerably between the 4 subpopulations (My1, My2, My3, My4) in Atlantic salmon liver (Taylor et al. 2022). Each ohnolog pair arising through 4R includes one *csf1r* copy that was virtually silenced across all myeloid populations (Fig. 2a), and another copy expressed in at least 2 of the myeloid populations. The 2 *csf1r* copies originating during 3R have diverged in expression in a cell type specific manner. Specifically, one *csf1r* ohnolog was expressed only in My1 and My2 (annotated as monocytes/macrophages; Taylor et al. 2022), with highest expression in My1, while the other ohnolog was expressed in My2, My3, and My4 with the highest expression in My2 (My3 and My4 annotated as dendritic cells; Taylor et al. 2022).

### Differences in Ohnolog Expression Between Immune Cell Subtypes

In the above analysis of ohnolog pair expression in the 5 major liver cell types, we aggregated all immune cells into a single population. This approach overlooks potential variation among immune subpopulations, such as the distinct *csf1r* expression patterns observed within myeloid cells. To account for this heterogeneity, we classified immune cells into 4 subtypes: B cells, T cells, natural killer-like cells, and myeloid cells (as detailed in Taylor et al. 2022). In cases where heterogeneity was previously reported within these immune cell subtypes (after Taylor et al. 2022; e.g. My1-My4 highlighted above and in Fig. 2a), we combined different subpopulations due to the small number of available nuclei in some cases, thus providing more statistical power for our tests. We then repeated the differential expression analysis of 4R ohnolog pairs in each immune cell subtype, opting not to downsample population sizes due to the limited number of nuclei available in each subtype.

Across the 4 immune cell subtypes, we identified 591 differentially expressed ohnolog pairs (Fig. 2c), an increase of 116 pairs compared with the analysis of the aggregated immune population (Fig. 1c). This increase was observed despite the reduced statistical power in individual subtypes stemming from smaller nuclei transcriptome counts compared with the global analysis. Differential expression of ohnolog pairs was predominantly confined to specific immune subtypes, rather than being uniformly distributed across all immune cells (Fig. 2c).

Although we observed significant variation in the number of differentially expressed ohnolog pairs across cell types, we refrain from making direct comparisons between immune cell types due to the differences in statistical power arising from disparities in population sizes. Nevertheless, it is clear that the differential expression of ohnologs is strongly cell type specific within the immune compartment.

### Cell-resolved Changes in Ohnolog Response to Infection

Transcriptomic studies of ohnolog expression patterns have typically focused on differences in unstimulated tissues or cell types, as demonstrated in the previous section of this study. Less explored is the extent to which ohnolog expression varies across cell types in response to physiological changes such as infection. To address this knowledge gap, we analyzed ohnolog expression responses 24 h following infection with the bacterial pathogen *Aeromonas salmonicida*. This immune challenge was previously shown to cause widespread remodeling of gene expression across different liver cell types (Taylor et al. 2022), but the previous analysis ignored ohnolog differences.

**Fig. 3.**
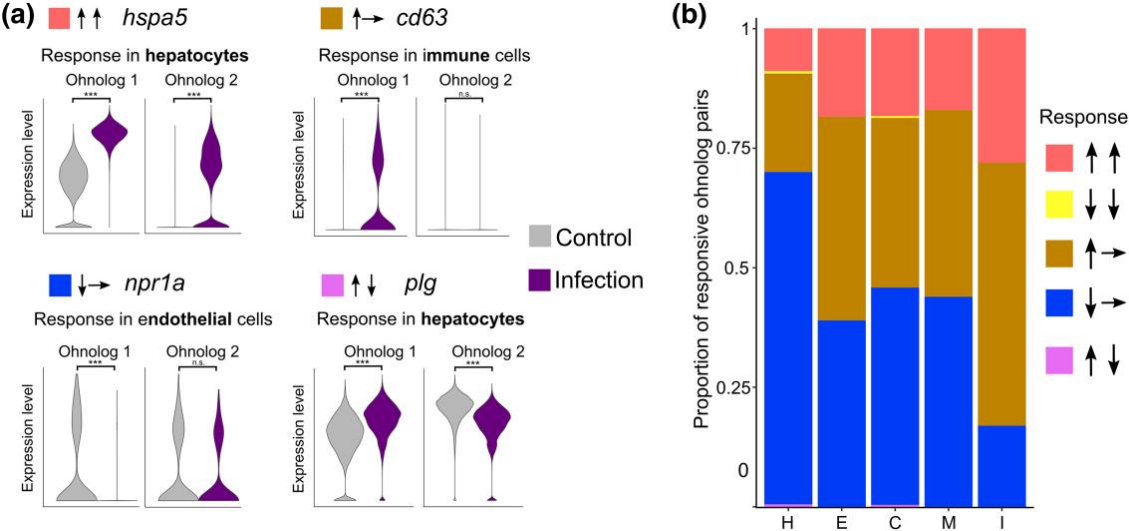
a) Examples of ohnologs arising from the 4R WGD that showed cell-specific expression responses to Aeromonas salmonicida infection: ohnologs for *hspa5*, encoding heat shock protein family A (Hsp70) member 5 (ohnolog 1: ENSSSAG00000067344; ohnolog 2: ENSSSAG00000068931) have different expression levels in hepatocytes in healthy liver and are both upregulated in response to infection; ohnologs for *cd63*, encoding lysosome-associated membrane protein 3 (ohnolog 1: ENSSSAG00000043967; ohnolog 2: ENSSSAG00000044857) show similar expression levels in immune cells in healthy liver, but only a single ohnolog is upregulated in response to infection; a single ohnolog of *npr1a*, encoding natriuretic peptide receptor 1a ohnolog is downregulated in response to infection in endothelial cells (ohnolog 1: ENSSSAG00000071305; ohnolog 2: ENSSSAG00000044690); plg, encoding plasminogen (ohnolog 1: ENSSSAG00000048657; ohnolog 2: ENSSSAG00000064000) offers a rare example of ohnologs showing opposite responses to infection, occurring in hepatocytes. Asterisks (***) denote statistical significance at *P* < 0.001. b) The response patterns of ohnolog pairs to infection in each of the 5 liver cell types. The categories distinguish both the direction of change of the members of each ohnolog pair (up ↑ or down ↓), and whether both members respond in unison (↑↑ and ↓↓) or not (↑→, ↓→, ↑↓). Colors correspond to the colors used in part a.

We categorized the response of ohnolog pairs into 3 distinct patterns: (1) a coordinated response (both genes responding significantly in the same direction), (2) a significant response in only one gene of the ohnolog pair, and (3) opposing significant responses between the 2 genes comprising an ohnolog pair (Fig. 3a).

Ohnolog response patterns were found to vary markedly across cell types. In hepatocytes, the predominant response was the downregulation of one gene in each ohnolog pair, while in immune cells, the upregulation of one member was most common (Fig. 3b). The remaining 3 cell types exhibited roughly equal proportions of pairs with one member upregulated or downregulated. In immune cells, 30% of ohnolog pairs showed coordinated upregulation of both members in response to infection, higher than the other cell types (supplementary table S5, Supplementary Material online). Hepatocytes displayed the highest proportion of ohnolog pairs (90%) with a response limited to a single member, whereas immune cells exhibited the lowest proportion (70%) (supplementary tables S6 and S7, Supplementary material online). Coordinated downregulation of both ohnologs in each pair was rare, and opposite response patterns were nearly absent across all cell types, with only a few instances detected in hepatocytes and cholangiocytes.

We further asked if differences could be observed in the proportion of responsive genes in each cell type that were members of ohnolog pairs or singletons. However, no statistically significant differences were observed for any cell type (supplementary fig. S1, Supplementary Material online).

## Discussion

Our study reveals cell type specific divergence in the expression of ohnologs retained from WGD events in fish evolution, with a focus on the salmonid specific WGD. Many past studies have compared ohnolog expression dynamics using bulk transcriptomic approaches. However, this approach obscures much of the complexity in ohnolog expression divergence shown here to occur in a cell-specific manner in Atlantic salmon liver. This was clearly illustrated within the immune cell compartment, where treating immune cell populations separately resulted in the identification of many more differentially expressed ohnolog pairs compared with treating the immune cells as a single population. It was also notable that, despite the relatively spare sequencing of the transcriptome of each nucleus in snRNA-seq, the proportion of ohnolog pairs found to be differentially expressed across all nuclei (11.2%) was comparable to the number found using the much deeper bulk RNA-seq data (12.6%). Using *cfs1r* as an example, we further illustrate the challenges that can be posed by the retention of multiple co-orthologous copies of classic marker genes commonly used to identify vertebrate cell lineages, when such markers have evolved cell type-specific expression. As such, our results indicate that future studies on gene expression evolution following WGD should adopt single cell approaches where possible, while single-cell transcriptomics studies in salmonids (and other species with recent WGD events) must consider all retained paralogous genes when choosing and interpreting marker genes used to annotate different cell types.

The divergence of ohnolog pair expression levels varied across Atlantic salmon liver cell types, being the highest in hepatocytes and lowest in immune cells. We speculate these results reflect differences in evolutionary constraint across cell types. Past work showed strong variation in the extent of orthologous gene expression similarity across mammalian tissues (Cardoso-Moreira et al. 2019), where the liver transcriptome was shown to be among the most rapidly evolving among a panel of tissues. Given that the liver is dominated by hepatocytes, it makes sense that this past bulk study's conclusions were largely explained by genes expressed in these cells. Conversely, the high conservation of ohnolog expression levels in immune cells implies stronger selection to maintain the function of both ohnologs in a pair. The higher proportion of ohnologs showing coordinated upregulation to bacterial infection in immune cells compared with other cell types is also consistent with selection to maintain the function of both ohnologs. Given that this study was limited to one tissue, more work is needed to understand variability in evolutionary constraints acting on ohnolog expression across a fuller range of tissue samples and hence cell types. Moreover, further work is needed to understand if evolutionary constraints acting on ohnolog expression across cell types are coupled to that acting on orthologs across species or whether functional redundancy introduced by WGD influences this relationship.

Ohnolog pairs were frequently observed to respond to bacterial infection with differences across cell types. The most common response for all cell types was for one member of an ohnolog pair to respond to the infection, consistent with widespread functional divergence, which could involve loss of ancestral regulatory elements responsible for transcriptional upregulation in one ohnolog. Considering a recent bulk transcriptomics study of liver ohnolog expression in salmonids, which found downregulation of one ohnolog was widespread (by far the most common expression fate) (Gillard et al. 2021), this interpretation seems more likely than widespread gains of expression responses to infection via neofunctionalization. Hepatocytes showed the highest proportion of ohnolog pairs where only one copy was responsive to infection, consistent with reduced selective pressure to maintain the ancestral response. Conversely, the higher proportion of ohnolog pairs retaining coordinated upregulation in immune cells following infection suggests selection to maintain regulatory elements responsive to bacterial stimulation. However, our data make it impossible to know whether both upregulated ohnologs are performing the same immunological function, as the role of the encoded proteins could have changed during salmonid evolution.

While evidencing the importance of cell type variation for understanding gene expression evolution post-WGD, there are limits to the conclusions that can be drawn from this study, not least because of our focus on a single tissue in a single species. While we focussed on the salmonid 4R WGD event in our global analysis, our results on *cfs1r*, alongside previous studies (Shafer et al. 2022), demonstrate the importance of cell-specific changes in gene expression following the teleost 3R WGD event, where there has been a greater period of evolution for ohnologs to diverge or specialise in cellular functions. This is an important area for future work, alongside considerations surrounding whether ohnolog cell-specific expression evolution is coupled across repeated WGD events (e.g. 3R and 4R), and whether ohnolog cell-specific expression evolution is distinct for other classes of gene duplicates not retained from WGD events. Since the basis for the observed expression changes following 4R is likely to involve transcriptional regulation, future work could seek to identify cell-resolved changes in epigenetic regulation, for example using single-cell ATAC-seq (Baek and Lee 2020), to define the role of regulatory elements as a driver for changes in ohnolog expression. Perhaps most importantly, the lack of an outgroup prevents us from inferring the ancestral state of ohnolog expression, hindering interpretations of the directionality of changes in gene expression and therefore inferences on processes including neofunctionalization and subfunctionalisation. Thus, future comparative single cell omics spanning different fish taxa, ideally using the Esociformes as an outgroup to 4R (Lien et al. 2016; Fig. 3), will be required to confidently resolve the cell-specific evolutionary fates of salmonid ohnologs.

## Materials and Methods

### Summary of snRNA-Seq Dataset

The snRNA-seq data were from a published study on Atlantic salmon liver (Taylor et al. 2022). Briefly, *n* = 4 samples were used, 2 from healthy control Atlantic salmon and 2 from fish sampled 24 h post-injection with the bacteria *Aeromonus salmonicida*. The snRNA-Seq libraries were constructed using 10 × Genomics technology (Taylor et al. 2022). Generation of the cell count matrix was conducted by mapping to the ICSASG_v2 reference genome assembly (Ensembl release 104) using STARSolo v2.7.7a and quality control and downstream analysis was performed with Seurat v4 (Hao et al. 2021). Cell annotation was performed by utilizing existing knowledge of marker genes to identify cell types. Full methods are described in the original publication (Taylor et al. 2022).

### Ohnolog Analyses

Ohnologs retained from the salmonid-specific WGD were obtained from a previous publication (Bertolotti et al. 2020). NCBI gene identifiers from that study were converted to Ensembl identifiers using the biomaRt (Durinck et al. 2009) “getBM” function (using the May 2021 Ensembl archive), with any genes lacking one-to-one correspondence between the 2 annotations excluded from analysis.

To identify cell-resolved differential expression between ohnolog pairs, the Seurat v3 R package (Hao et al. 2021) was used with the control samples. To ensure equivalent statistical power across cell types, each of the 5 cell clusters were randomly downsampled to match the size of the smallest cluster, resulting in 428 nuclei for comparison of the 5 main cell types. For the immune cell subtype comparison, downsampling was not performed, due to the low number of nuclei in some of the subpopulations.

Differential expression between members of each ohnolog pair was determined by performing a Wilcoxon rank-sum test (adjusted *P*-value < 0.05, log2 fold change > 0.25) within each cluster. In this test, the expression of one member of each ohnolog pair was removed from a cluster and compared against the same cluster with the expression of the other member removed, treating both members of each ohnolog pair as the same gene.

To investigate differential responses of ohnologs to the bacterial infection, the full dataset of 2 control fish versus 2 infected fish was used (Taylor et al. 2022). The Seurat function FindAllMarkers was used for each population in turn to perform DGE tests (Wilcoxon rank sum, log2fc = 0.25, adjusted *P*-value < 0.05) between cells from the control and infected fish. For each population, all ohnolog pairs were classified into the 5 response groups depicted in Fig. 3 according to the results of the test.

Fisher exact tests (supplementary tables S1 to S7, Supplementary Material online) were conducted in R using the package Rstatix and command pairwise_fisher_test.

### Bulk RNA-seq Differential Gene Expression Test

TPM counts from an existing RNA-seq dataset in Atlantic salmon liver (Gillard et al. 2021;  *n* = 8) were downloaded and gene names mapped to the Ensembl IDs used in this study. DESeq2 was used to conduct a differential gene expression between a count matrix of the TPM counts of first member of each ohnolog pair against a count matrix of the TPM values of the other member. Thresholds of log2FoldChange > 0.25 and adjusted *P*-value < 0.05 were chosen to match the snRNA-seq.

### Phylogenetic Analysis for *csf1r* Gene Family

The amino acid sequences of the “canonical” version of *csf1r* genes, and all predicted orthologs, were downloaded from Ensembl (release 112) for the following species: *Lepisosteus oculatus*, *Danio rerio, Tetraodon nigroviridis, Esox Lucius*, and *S. salar.* Sequences were aligned with MAFFT version 7 (Katoh et al. 2019; default settings) and the gene tree built using maximum likelihood with IQ-TREE (Minh et al. 2020), using the best fitting amino acid substitution model selected by ModelFinder (Kalyaanamoorthy et al. 2017), which was JTT + G4 + I. Bootstrap support was estimated using the UFBoot method (Hoang et al. 2018). The tree was rooted using *L. oculatus* as an outgroup to the teleost 3R WGD event.

## Supplementary Material

evaf076_Supplementary_Data

## Data Availability

No new data were generated in support of this research. The publically available data underlying this article are available in the GEO database (https://www.ncbi.nlm.nih.gov/geo/). Accession: GSE207655.
