# Supplementary material for: Cell Type Resolved Expression of Duplicate Genes Retained From Whole Genome Duplication in Atlantic salmon
Source: Genome Biol Evol. 2025 Apr 30;17(5):evaf076. doi: 10.1093/gbe/evaf076 (PMC12082034; doi:10.1093/gbe/evaf076)
Supplement: evaf076_Supplementary_Data [file evaf076_supplementary_data.docx]

**Supplementary Table 1.** Fisher’s Exact Tests comparing the number of ohnolog pairs showing cell type-specific differential expression with respect to the number of ohnolog pairs expressed in the dataset, across all combinations of the five major cell types in Atlantic salmon liver. Summarised in Fig. 1C Upset plot, where letters summarise below tests.

| **Cell type 1** | **Cell type 2** | **Cell type 1** *Ohnolog pairs expressed in dataset (not uniquely differentially expressed in cell type)* | **Cell type 1** *Ohnolog pairs uniquely differentially expressed in cell type* | **Cell type 2**  *Ohnolog pairs expressed in dataset (not uniquely differentially expressed in cell type)* | **Cell type 2**  *Ohnolog pairs uniquely differentially expressed in cell type* | **Adjusted p-value** | **Significance** |
| --- | --- | --- | --- | --- | --- | --- | --- |
| Hepatocytes | Cholangiocytes | 8001 | 544 | 8366 | 179 | 2.13e-44 | **** |
| Hepatocytes | Mesenchymal | 8001 | 544 | 8372 | 173 | 2.53e-46 | **** |
| Hepatocytes | Endothelial | 8001 | 544 | 8344 | 201 | 6.83e-38 | **** |
| Hepatocytes | Immune | 8001 | 544 | 8432 | 113 | 5.82e-70 | **** |
| Cholangiocytes | Mesenchymal | 8366 | 179 | 8372 | 173 | 1.00E+00 | ns |
| Cholangiocytes | Endothelial | 8366 | 179 | 8344 | 201 | 1.00E+00 | ns |
| Cholangiocytes | Immune | 8366 | 179 | 8432 | 113 | 1.17E- 3 | ** |
| Mesenchymal | Endothelial | 8372 | 173 | 8344 | 201 | 1.00E+00 | ns |
| Mesenchymal | Immune | 8372 | 173 | 8432 | 113 | 4.16E-03 | ** |
| Endothelial | Immune | 8344 | 201 | 8432 | 113 | 6.16E-06 | **** |

**Supplementary Table 2.** Fisher’s Exact Tests comparing the number of ohnolog pairs that show differential expression with respect to the number of ohnolog pairs expressed in the dataset, across all combinations of the five major cell types in Atlantic salmon liver. Summarised in Fig. 1C sub-panel ‘Total differentially expressed pairs’, where letters summarise below tests.

| **Cell type 1** | **Cell type 2** | **Cell type 1** *Ohnolog pairs expressed in dataset (not differentially expressed)* | **Cell type 1** *Ohnolog pairs differentially expressed in cell type (including shared with other cell types)* | **Cell type 2**  *Ohnolog pairs expressed in dataset (not differentially expressed* | **Cell type 2**  *Ohnolog pairs differentially expressed in cell type (including shared with other cell types)* | **Adjusted p-value** | **Significance** |
| --- | --- | --- | --- | --- | --- | --- | --- |
| Hepatocytes | Cholangiocytes | 7300 | 1245 | 7852 | 693 | 8.56E-40 | **** |
| Hepatocytes | Mesenchymal | 7300 | 1245 | 7914 | 631 | 1.13eE-50 | **** |
| Hepatocytes | Endothelial | 7300 | 1245 | 8070 | 746 | 8.30eE-32 | **** |
| Hepatocytes | Immune | 7300 | 1245 | 8070 | 475 | 7.63E-87 | **** |
| Cholangiocytes | Mesenchymal | 7852 | 693 | 7914 | 631 | 8.09E-01 | ns |
| Cholangiocytes | Endothelial | 7852 | 693 | 8070 | 746 | 1.00E+00 | ns |
| Cholangiocytes | Immune | 7852 | 693 | 8070 | 475 | 4.23E-10 | **** |
| Mesenchymal | Endothelial | 7914 | 631 | 8070 | 746 | 1.35E-02 | * |
| Mesenchymal | Immune | 7914 | 631 | 8070 | 475 | 1.39E-05 | **** |
| Endothelial | Immune | 8070 | 746 | 8070 | 475 | 8.34E-15 | **** |

**Supplementary Table 3.** Fisher’s Exact Tests comparing the number of ohnolog pairs that show differential expression with respect to the number of ohnolog pairs expressed in each cell type, across all combinations of the five major cell types in Atlantic salmon liver. Summarised in Fig. 1C sub-panel ‘Proportion of all expressed pairs showing differential expression’, where letters summarise below tests.

| **Cell type 1** | **Cell type 2** | **Cell type 1**  *Ohnolog pairs expressed in cell type (not differentially expressed)* | **Cell type 1**  *Ohnolog pairs differentially expressed in cell type (including shared with other cell types)* | **Cell type 2**  *Ohnolog pairs expressed in cell type (not differentially expressed)* | **Cell type 2**  *Ohnolog pairs differentially expressed in cell type (including shared with other cell types)* | **Adjusted p-value** | **Significance** |
| --- | --- | --- | --- | --- | --- | --- | --- |
| Hepatocytes | Cholangiocytes | 6398 | 1245 | 6406 | 693 | 4.66e-31 | **** |
| Hepatocytes | Mesenchymal | 6398 | 1245 | 6173 | 631 | 1.32e-35 | **** |
| Hepatocytes | Endothelial | 6398 | 1245 | 5966 | 746 | 2.29e-18 | **** |
| Hepatocytes | Immune | 6398 | 1245 | 6490 | 475 | 6.83e-72 | **** |
| Cholangiocytes | Mesenchymal | 6406 | 693 | 6173 | 631 | 1.00E+00 | ns |
| Cholangiocytes | Endothelial | 6406 | 693 | 5966 | 746 | 9.56e-02 | ns |
| Cholangiocytes | Immune | 6406 | 693 | 6490 | 475 | 2.80 e-09 | **** |
| Mesenchymal | Endothelial | 6173 | 631 | 5966 | 746 | 4.19e-03 | ** |
| Mesenchymal | Immune | 6173 | 631 | 6490 | 475 | 1.31e-06 | **** |
| Endothelial | Immune | 5966 | 746 | 6490 | 475 | 1.10e-17 | **** |

**Supplementary Table 4.** Fisher’s Exact Tests comparing the number of ohnolog pairs that show differential expression with respect to the number of ohnolog pairs expressed in each cell type, across all combinations of the five major cell types in Atlantic salmon liver. Summarised in Fig. 1C sub-panel ‘Proportion of differential expression unique to cell type’, where letters summarise below tests.

| **Cell type 1** | **Cell type 2** | **Cell type 1** *Ohnolog pairs differentially expressed (not cell type specific)* | **Cell type 1** *Ohnolog pairs differentially expressed (cell-specific)* | **Cell type 2**  *Ohnolog pairs differentially expressed (not cell type specific)* | **Cell type 2**  *Ohnolog pairs differentially expressed (cell-specific)* | **Adjusted p-value** | **Significance** |
| --- | --- | --- | --- | --- | --- | --- | --- |
| Hepatocytes | Cholangiocytes | 701 | 544 | 514 | 179 | 3.49e-14 | **** |
| Hepatocytes | Mesenchymal | 701 | 544 | 458 | 173 | 4.79e-11 | **** |
| Hepatocytes | Endothelial | 701 | 544 | 545 | 201 | 4.86e-13 | **** |
| Hepatocytes | Immune | 701 | 544 | 362 | 113 | 1.26e-13 | **** |
| Cholangiocytes | Mesenchymal | 514 | 179 | 458 | 173 | 1.00E+00 | ns |
| Cholangiocytes | Endothelial | 514 | 179 | 545 | 201 | 1.00E+00 | ns |
| Cholangiocytes | Immune | 514 | 179 | 362 | 113 | 1.00E+00 | ns |
| Mesenchymal | Endothelial | 458 | 173 | 545 | 201 | 1.00E+00 | ns |
| Mesenchymal | Immune | 458 | 173 | 362 | 113 | 1.00E+00 | ns |
| Endothelial | Immune | 545 | 201 | 362 | 113 | 1.00E+00 | ns |

**Supplementary Table 5.** Fisher’s Exact Test comparing the proportion of ohnolog pairs that were both upregulated in response to *Aeromonus salmonicida* infection between the five major cell types in Atlantic salmon liver.

| **Cell type 1** | **Cell type 2** | **Cell type 1** *All differentially responsive* *ohnolog pairs* | **Cell type 1** *Both* *ohnolog pairs upregulated* | **Cell type 2**  *All differentially responsive ohnolog pairs* | **Cell type 2** *Both* *ohnolog pairs upregulated* | **Adjusted p-value** | **Significance** |
| --- | --- | --- | --- | --- | --- | --- | --- |
| Hepatocytes | Cholangiocytes | 603 | 54 | 195 | 36 | 4.86E-03 | ** |
| Hepatocytes | Mesenchymal | 603 | 54 | 251 | 46 | 1.56E-03 | ** |
| Hepatocytes | Endothelial | 603 | 54 | 246 | 42 | 8.12E-03 | ** |
| Hepatocytes | Immune | 603 | 54 | 171 | 48 | 1.94E-08 | **** |
| Cholangiocytes | Mesenchymal | 195 | 36 | 251 | 46 | 1.00E+00 | ns |
| Cholangiocytes | Endothelial | 195 | 36 | 246 | 42 | 1.00E+00 | ns |
| Cholangiocytes | Immune | 195 | 36 | 171 | 48 | 1.37E-01 | ns |
| Mesenchymal | Endothelial | 251 | 46 | 246 | 42 | 1.00E+00 | ns |
| Mesenchymal | Immune | 251 | 46 | 171 | 48 | 1.16E-01 | ns |
| Endothelial | Immune | 246 | 42 | 171 | 48 | 4.76E-02 | * |

**Supplementary Table 6**. Fisher’s Exact Test comparing the proportion of ohnolog pairs where a single ohnolog was upregulated in response to *Aeromonus* *salmonicida* infection (and the other showed no response) between the five major cell types in Atlantic salmon liver.

| **Cell type 1** | **Cell type 2** | **Cell type 1** *All differentially responsive* o*hnolog pairs* | **Cell type 1** *Pairs with* *single* *ohnolog upregulated* | **Cell type 2**  *All differentially responsive ohnolog pairs* | **Cell type 2** *Pairs with* *single* *ohnolog upregulated* | **Adjusted p-value** | **Significance** |
| --- | --- | --- | --- | --- | --- | --- | --- |
| Hepatocytes | Cholangiocytes | 603 | 124 | 195 | 83 | 3.45E-08 | **** |
| Hepatocytes | Mesenchyme | 603 | 124 | 251 | 89 | 5.93E-05 | **** |
| Hepatocytes | Endothelial | 603 | 124 | 246 | 96 | 5.30E-07 | **** |
| Hepatocytes | Immune | 603 | 124 | 171 | 94 | 2.59E-16 | **** |
| Cholangiocytes | Mesenchyme | 195 | 83 | 251 | 89 | 4.26E-01 | ns |
| Cholangiocytes | Endothelial | 195 | 83 | 246 | 96 | 9.16E-01 | ns |
| Cholangiocytes | Immune | 195 | 83 | 171 | 94 | 8.44E-02 | ns |
| Mesenchyme | Endothelial | 251 | 89 | 246 | 96 | 9.16E-01 | ns |
| Mesenchyme | Immune | 251 | 89 | 171 | 94 | 5.50E-03 | *** |
| Endothelial | Immune | 246 | 96 | 171 | 94 | 7.00E-03 | ** |

**Supplementary Table 7**. Fisher’s Exact Test comparing the proportion of ohnolog pairs where a single ohnolog was downregulated in response to *Aeromonus salmonicida* infection (and the other showed no response) between the five major cell types in Atlantic salmon liver.

| **Cell type 1** | **Cell type 2** | **Cell type 1** *All differentially responsive* o*hnolog pairs* | **Cell type 1** *Pairs with* *single* *ohnolog* *downregulated* | **Cell type 2**  *All differentially responsive ohnolog pairs* | **Cell type 2** *Pairs with* *single* *ohnolog downregulated* | **Adjusted p-value** | **Significance** |
| --- | --- | --- | --- | --- | --- | --- | --- |
| Hepatocytes | Cholangiocytes | 603 | 419 | 195 | 76 | 5.62E-13 | **** |
| Hepatocytes | Mesenchyme | 603 | 419 | 251 | 114 | 5.90E-10 | **** |
| Hepatocytes | Endothelial | 603 | 419 | 246 | 108 | 5.90E-11 | **** |
| Hepatocytes | Immune | 603 | 419 | 171 | 29 | 1.53E-34 | **** |
| Cholangiocytes | Mesenchyme | 195 | 76 | 251 | 114 | 5.34E-01 | ns |
| Cholangiocytes | Endothelial | 195 | 76 | 246 | 108 | 6.62E-01 | ns |
| Cholangiocytes | Immune | 195 | 76 | 171 | 29 | 1.26E-05 | **** |
| Mesenchyme | Endothelial | 251 | 114 | 246 | 108 | 7.87E-01 | ns |
| Mesenchyme | Immune | 251 | 114 | 171 | 29 | 4.77E-09 | **** |
| Endothelial | Immune | 246 | 108 | 171 | 29 | 2.20E-08 | ***** |


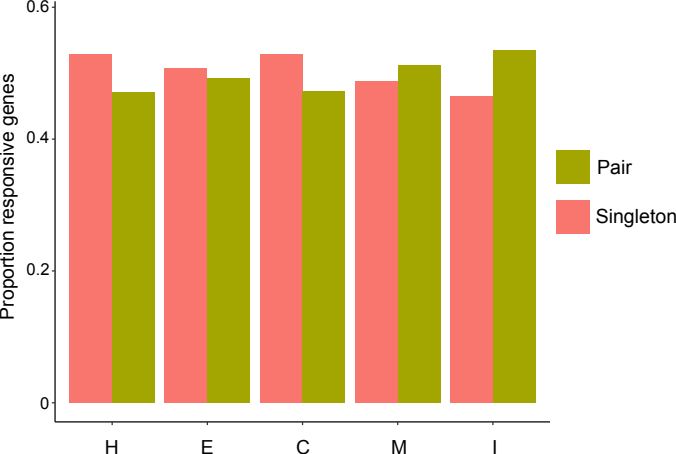


**Supplementary Figure 1**: The proportion of genes in each cell-type exhibiting expression changes in response to *A. salmonida* infection that are categorised as ohnolog pairs (either or both gene in the pair responsive) or singletons. All pairwise differences between cell-types were found to be non-significant (Fisher exact test).
